# The scientific basis for secondary prevention of coronary artery disease: recent contributions from the Netherlands

**DOI:** 10.1007/s12471-020-01450-w

**Published:** 2020-08-11

**Authors:** H. T. Jørstad, M. Snaterse, N. ter Hoeve, M. Sunamura, R. Brouwers, H. Kemps, W. J. M. Scholte op Reimer, R. J. G. Peters

**Affiliations:** 1 Department of Cardiology, Heart Center, Amsterdam Cardiovascular Sciences, Amsterdam UMC, University of Amsterdam, Amsterdam, The Netherlands; 2 ACHIEVE Centre of Applied Research, Faculty of Health, Amsterdam University of Applied Sciences, Amsterdam, The Netherlands; 3 Capri Cardiac Rehabilitation, Rotterdam, The Netherlands; 4 Department of Rehabilitation Medicine, Erasmus MC, University Medical Centre, Rotterdam, The Netherlands; 5 Department of Cardiology, Máxima Medical Center, Eindhoven, The Netherlands; 6 Eindhoven University of Technology, Eindhoven, The Netherlands

**Keywords:** Secondary prevention, Coronary artery disease, Randomised controlled trials, Lifestyle, Risk factors

## Abstract

While the beneficial effects of secondary prevention of cardiovascular disease are undisputed, implementation remains challenging. A gap between guideline-mandated risk factor targets and clinical reality was documented as early as the 1990s. To address this issue, research groups in the Netherlands have performed several major projects. These projects address innovative, multidisciplinary strategies to improve medication adherence and to stimulate healthy lifestyles, both in the setting of cardiac rehabilitation and at dedicated outpatient clinics. The findings of these projects have led to changes in prevention and rehabilitation guidelines.

## Dutch contribution to the field

- The web-based tool www.u-prevent.com and the underlying models have been seminal in cardiovascular risk prediction and estimation of treatment effects.
- Nurse-coordinated programs (RESPONSE‑1 and RESPONSE‑2 randomised trials) improve risk factor control, medication optimisation, and reduce non-cardiac emergency room presentation.
- Nurse-coordinated referral to community-based lifestyle programs is successful in improving lifestyle-related risk factors, especially for weight reduction.
- Adding face-to-face physical activity counselling as an extension to standard cardiac rehabilitation results in improvements in daily step counts (OPTICARE randomised trial).
- Cardiac telerehabilitation (FIT@Home randomised trial) is non-inferior to conventional cardiac rehabilitation, and is cost-effective.

## Introduction

While the beneficial effects of secondary prevention of cardiovascular disease (CVD) are undisputed, implementation remains challenging. A gap between guideline-mandated risk factor targets and clinical reality was documented as early as the 1990s. Both medical therapy of biometric risk factors and interventions to modify lifestyle have consistently been shown to lag behind increasingly stringent guidelines [1–3]. To address this issue, research groups in the Netherlands have performed several major projects. First, using data from landmark trials, various models have been developed to predict the effect of different treatments for individual patients in terms of absolute risk, integrated into a web-based tool (www.u-prevent.com). Second, large randomised trials have been performed to investigate interventions to improve the quality of secondary prevention. These trials address innovative, multidisciplinary strategies to improve medication adherence and to stimulate healthy lifestyles, both in the setting of cardiac rehabilitation and at dedicated outpatient clinics. The findings of these projects have led to changes in prevention and rehabilitation guidelines, and the main findings, implications and opportunities for further research are outlined in this paper.

## U-Prevent prediction models

In an increasingly complex field of treatment modalities and strategies for patients with CVD, decisions on initiation or intensification of preventive treatment can be assisted by assessing individual anticipated clinical benefit, derived from prediction models. Key models have been integrated into an easy-to-use web-based tool (www.u-prevent.com). These models cover a large variety of patients, including medium-term risk estimation for patients with established CVD (SMART) [4], and in individuals aged >70 years with and without established CVD [5], in addition to lifetime risk estimation and treatment effect in patients with a broad spectrum of CVD (SMART-REACH) [6], and diabetes mellitus type 2 (DIAL) [7]. Such prediction models are rapidly becoming important tools for clinicians aiming to personalise preventive therapy in patients with CVD.

## Experiences from the randomised RESPONSE 1 and 2 trials

While complex interventions have been shown to be moderately successful in secondary prevention, implementation of such programs outside research settings has been limited (for example the large-scale EuroAction trial) [8]. The Randomised Evaluation of Secondary Prevention by Outpatient Nurse SpEcialists (RESPONSE) 1 (2006–2009) trial (*n* = 754) was designed to quantify the impact of a practical, hospital-based nurse coordinated prevention program, integrated into the routine clinical care of patients in the first year after an acute coronary syndrome (ACS) [9]. The nurse-coordinated program consisted of up to four outpatient clinic visits focusing on (1) healthy lifestyle; (2) biometric risk factors; and (3) medication adherence, on top of usual care. At 12-months follow-up, the estimated overall impact on cardiovascular risk was a 17% relative reduction in patients in the intervention group as compared with patients receiving only usual care (*p* = 0.021). This difference was largely driven by intensified medication titration [10], with better treatment to target levels for LDL cholesterol and blood pressure. This was associated with slight increases in health-related quality of life, and a reduction in depressive symptoms in patients randomised to the nurse-coordinated program [11]. There were only slight improvements in self-reported lifestyle parameters such as physical activity and diet, and no improvements in smoking cessation or body mass index. Surprisingly, a decrease in emergency room presentations/readmissions was observed, in favour of individuals attending the nurse-coordinated program (86 vs 132, *p* = 0.023), potentially reflecting the counselling part of the nurse-coordinated program and the positive changes in quality of life and confidence, and reduced depressive symptoms. We therefore recommended that nurse-coordinated programs should be part of the usual care of patients with an ACS, a recommendation which was adopted by the ESC prevention guidelines (level of evidence IIa) [1].

Based on the findings from RESPONSE‑1, the RESPONSE‑2 trial was designed (2013–2016) [12]. The RESPONSE‑2 trial continued with the concept of the central role of a coordinating nurse specialist but focused specifically on lifestyle modification and partner participation. To increase the probability of successful lifestyle modification, the role of the nurse in RESPONSE‑2 was to identify risk profiles, to motivate patients and to refer both patients and their partners to readily available community-based commercial lifestyle interventions (weight reduction, smoking cessation and physical activity programs). A total of 824 patients with ACS or coronary revascularisation were randomised to either usual care, this time including RESPONSE‑1 nurse visits, or to the intervention group, which consisted of usual care and RESPONSE‑1 visits, plus coordinated referrals to external lifestyle programs for patients and their partners, if applicable. Due to the complex interplay of risk factors and risk factor modification in secondary prevention, a composite overall outcome measure was defined to take not only improvement of lifestyle-related risk factors into account, but also deterioration. Thus, a strict definition of successful lifestyle modification was used for the primary outcome: a clinically relevant improvement in ≥1 qualifying lifestyle-related risk factor at 12-months follow-up (weight, smoking, physical activity) without deterioration in the other risk factors. At 12-months follow-up, 37% of patients in the intervention group versus 26% in the usual care group (*p* = 0.002) reached the primary outcome, i.e. showed a net improvement in ≥1 lifestyle-related risk factor. The effect was the most prominent in weight reduction (≥5% weight reduction was 27% for the intervention vs. 14% for usual care, *p* < 0.001). Active partner participation in the intervention group was associated with a significantly greater success rate (46% in this subgroup reached the primary outcome), while the absence of a partner in the usual care group was associated with the lowest success rate (10%). These findings indicate that referral of patients with CVD and their partners to a comprehensive set of community-based lifestyle programs improves lifestyle-related factors more than guideline-based usual care alone [12].

## Optimising cardiac rehabilitation

Although cardiac rehabilitation has well-known benefits, current programs seem insufficient to improve physical activity [13, 14]. The OPTICARE (OPTImizing CArdiac Rehabilitation) research group aims to optimise cardiac rehabilitation with regard to adopting a healthy lifestyle. In the OPTICARE‑1 randomised controlled trial (RCT), the effects of extending cardiac rehabilitation with additional behavioural counselling (face-to-face group sessions or individual telephone sessions) were investigated in 914 ACS patients [15]. Compared with standard cardiac rehabilitation, adding face-to-face physical activity counselling resulted in additional improvement in daily step counts [16]. Furthermore, the face-to-face counselling resulted in an improved maintenance of physical fitness up to 12 months and in long-term reductions in fatigue [17]. The extra counselling sessions did not confer additional benefits with respect to blood pressure, cholesterol, or smoking. Patients largely reached the target levels for these risk factors following standard cardiac rehabilitation [15]. Telephone counselling did not provide additional benefits, emphasising the importance of face-to-face contact.

Selected subgroups, such as patients with obesity and women, seem less likely to profit from cardiac rehabilitation [18, 19]. In the OPTICARE XL RCT, the effectiveness of a novel cardiac rehabilitation program for obese patients is currently being investigated in 201 patients with coronary artery disease (CAD) or atrial fibrillation. The program includes small group participation, self-management, and physical training programs. Results from a pilot study (*n* = 17) showed that in the first 3 months, patients significantly improved their health-related quality of life by 24%, fitness by 15%, and reduced their BMI by 6%. Results of the full study are expected in 2021.

## Specific lessons

In both RESPONSE‑1 and RESPONSE‑2, the OPTICARE RCT [9, 12, 15], and in European survey data [20, 21], a highly stable rate of smoking cessation is observed of ~50% after an ACS or revascularisation. The majority of successful quitters quit during or immediately after hospitalisation [22], regardless of participation in smoking cessation programs. Immediate quitters even preferred not to attend smoking cessation programs but were highly motivated for other lifestyle programs [23]. The effect of an acute coronary event should therefore not be underestimated in motivating patients to stop smoking; however, hard-core smokers remain an important challenge.

Individual choices of lifestyle programs are not typically directly related to risk profiles. Individuals with overweight frequently chose physical activity programs instead of weight reduction programs, while ex-smokers frequently chose weight-reduction programs. Currently, most lifestyle modification programs select individuals based on the presence of a single specific risk factor. However, from a patient’s perspective it is preferable to comprehensively address all relevant lifestyle-related risk factors and to discuss a patient’s perspectives and preferences. Studies investigating personalisation of lifestyle interventions are therefore urgently needed.

The RESPONSE‑1 and RESPONSE‑2 trials demonstrated that risk profiles can be improved at 12 months after the index event. However, several challenges remain. First, the long-term sustainability of these improvements needs to be evaluated at longer follow-up. Three-year follow-up data from the RESPONSE‑2 trial are currently being analysed. Second, in spite of these successful outcomes, residual risk at 12 months is still considerable. With the arrival of new pharmacological agents targeting established risk factors (e.g. antithrombotics, anticoagulation, overweight, LDL reduction, and novel cholesterol-lowering strategies) and newly identified risk factors (e.g. inflammation and triglycerides), the complexity of secondary prevention will significantly increase. The costs of such strategies are high, and no study has yet investigated how these agents should be integrated into daily practice and in which combinations, with or without intensive lifestyle interventions. Therefore, there is a need for further secondary prevention trials to investigate how to implement value-based, patient-centred treatment strategies (Box 1).

### Box 1 Future research opportunities

- Consolidation of lifestyle modification at longer follow-up
- Implementation of value-based, patient-centred treatment strategies, i.e. personalisation
- Improve quality and outcomes of CR
- Further development of telerehabilitation

## E-health: cardiac telerehabilitation

In cardiac telerehabilitation (CTR), components of cardiac rehabilitation programs are offered outside the environment of the rehabilitation centre, using remote communication and devices to monitor parameters, such as heart rate and physical activity. This may reduce accessibility barriers (e.g. travelling), but may also increase patients’ self-management skills, resulting in sustainable behavioural change [24, 25]. In recent years, several studies have evaluated the effects of CTR. The FIT@Home study [26] was one of the first large RCTs investigating CTR versus centre-based cardiac rehabilitation [27, 28]. In total, 90 patients with CAD with low to moderate risk of recurrent CVD events were randomised to a 12-week program of either centre-based or home-based exercise training. The centre-based group participated in group-based training sessions (2 sessions of 45–60 min/week) based on continuous training at 75–80% maximal heart rate. The intervention group performed the same training program at home after three familiarisation sessions at the cardiac rehabilitation centre, using a heart rate monitor and uploading training data to a web application. Patients received weekly feedback by telephone from their physical therapist and were encouraged to continue using the heart rate monitor and web application after 12 weeks. After 1 year, there were no between-group differences for the primary outcomes of physical fitness (peakVO2) and objectively assessed physical activity [29]. Cost-effectiveness analysis showed that societal costs per patient were € 3160 lower for those in the home-based group, mainly driven by earlier work resumption. Although highly cost-effective, the FIT@Home intervention did not show superior results with respect to physical fitness or activity behaviour as compared with centre-based cardiac rehabilitation. Therefore, a second RCT was designed, aiming to demonstrate superior long-term effects of CTR with respect to exercise behaviour. The SmartCare-CAD study has randomised 300 patients with CAD to either centre-based or home-based training [30]. Unique to this trial is that patients are provided feedback not only on adherence to prescribed training sessions but also on daily energy expenditure, and the intervention includes relapse prevention and tailored patient goals. Results of the SmartCare-CAD trial are expected to be published later this year.

## Conclusion

Implementation remains a central issue in secondary prevention of CVD. For drug-related therapy this is challenging, and for changing lifestyle-related risk factors even more challenging. Only a minority of patients are able to successfully and permanently correct all relevant lifestyle issues. Both patient and caregiver factors impact the overall success rate and need to be targeted in ongoing and future investigations. Major developments are seen in individualising the estimation of the risk of recurrent events and in designing individual preventive programs, based on patients’ characteristics and preferences. In the actual programs, there have been important steps in involving the patient’s partner, addressing patient preferences and in the application of home-based or community-based programs, including remote monitoring and coaching. Given the significant benefits to patients if the cause of their disease is successfully treated, we should continue to make every effort to improve the efficacy of programs for secondary prevention of CAD.

## References

[1] Piepoli MF, Hoes AW, Agewall S, et al. 2016 European Guidelines on cardiovascular disease prevention in clinical practice: The Sixth Joint Task Force of the European Society of Cardiology and Other Societies on Cardiovascular Disease Prevention in Clinical Practice (constituted by representatives of 10 societies and by invited experts): Developed with the special contribution of the European Association for Cardiovascular Prevention & Rehabilitation (EACPR). Eur Heart J 2016;37:2315–81. 10.1093/eurheartj/ehw106 PMC4986030 27222591

[2] Eckel R, Jakicic J, Ard J, et al. 2013 AHA/ACC Guideline on Lifestyle Management to Reduce Cardiovascular Risk. J Am Coll Cardiol. 2014;63(25 Part B):2960–84. 10.1016/j.jacc.2013.11.003 24239922

[3] Stone N, Robinson J, Lichtenstein A, et al. 2013 ACC/AHA Guideline on the Treatment of Blood Cholesterol to Reduce Atherosclerotic Cardiovascular Risk in Adults. J Am Coll Cardiol. 2014;63(25 Part B):2889–934. 10.1016/j.jacc.2013.11.002 24239923

[4] Dorresteijn J, Visseren F, Wassink A, et al. SMART Study Group. Development and validation of a prediction rule for recurrent vascular events based on a cohort study of patients with arterial disease (2013). the SMART risk score. Heart.

[5] Stam-Slob M, Visseren F, Jukema J (2017). Personalized absolute benefit of statin treatment for primary or secondary prevention of vascular disease in individual elderly patients. Clin Res Cardiol.

[6] Kaasenbrood L, Bhatt DL, Dorresteijn J (2018). Estimated Life Expectancy Without Recurrent Cardiovascular Events in Patients With Vascular Disease: The SMART-REACH Model. J Am Heart Assoc.

[7] Berkelmans GFN, Gudbjörnsdottir S, Visseren FLJ (2019). Prediction of individual life-years gained without cardiovascular events from lipid, blood pressure, glucose, and aspirin treatment based on data of more than 500 000 patients with Type 2 diabetes mellitus. Eur Heart J.

[8] Wood DA, Kotseva K, Connolly S (2008). Nurse-coordinated multidisciplinary, family-based cardiovascular disease prevention programme (EUROACTION) for patients with coronary heart disease and asymptomatic individuals at high risk of cardiovascular disease: a paired, cluster-randomised controlled trial. Lancet.

[9] Jørstad HT, von Birgelen C, Alings AM (2013). Effect of a nurse-coordinated prevention programme on cardiovascular risk after an acute coronary syndrome: main results of the RESPONSE randomised trial. Heart.

[10] Snaterse M, Jørstad HT, Heiligenberg M (2017). Nurse-coordinated care improves the achievement of LDL-cholesterol targets through more intensive medication titration. Open Heart.

[11] Jørstad HT, Minneboo M, Helmes HJ (2016). Effects of a Nurse-Coordinated Prevention Programme on Health-Related Quality of Life and Depression in Patients with an Acute Coronary Syndrome: Results from the RESPONSE Randomised Controlled Trial. BMC Cardiovasc Disord.

[12] Minneboo M, Lachman S, Snaterse M (2017). Community-Based Lifestyle Intervention in Patients With Coronary Artery Disease: The RESPONSE 2 Trial. J Am Coll Cardiol.

[13] ter Hoeve N, Huisstede B, Stam H (2015). Does cardiac rehabilitation after an acute cardiac syndrome lead to changes in physical activity habits? Systematic review. Phys Ther.

[14] Ter Hoeve N, Sunamura M, van Geffen M (2017). Changes in Physical Activity and Sedentary Behavior During Cardiac Rehabilitation. Arch Phys Med Rehabil.

[15] Sunamura M, ter Hoeve N, van den Berg-Emons RJG (2018). Randomised controlled trial of two advanced and extended cardiac rehabilitation programmes. Heart.

[16] Ter Hoeve N, Sunamura M, Stam H (2018). Effects of two behavioral cardiac rehabilitation interventions on physical activity: A randomized controlled trial. Int J Cardiol.

[17] Ter Hoeve N, Sunamura M, Stam H (2019). A secondary analysis of data from the OPTICARE randomized controlled trial investigating the effects of extended cardiac rehabilitation on functional capacity, fatigue, and participation in society. Clin Rehabil.

[18] Minges KE, Strait KM, Owen N (2017). Gender differences in physical activity following acute myocardial infarction in adults: A prospective, observational study. Eur J Prev Cardiol.

[19] Martin BJ, Aggarwal SG, Stone JA (2012). Obesity negatively impacts aerobic capacity improvements both acutely and 1-year following cardiac rehabilitation. Obes (silver Spring).

[20] Kotseva K, De Backer G, De Bacquer D, et al. EUROASPIRE Investigators. Lifestyle and impact on cardiovascular risk factor control in coronary patients across 27 countries (2019). Results from the European Society of Cardiology ESC-EORP EUROASPIRE V registry. Eur J Prev Cardiol.

[21] Snaterse M, Deckers JW, Lenzen M, et al. for the EUROASPIRE investigators. Smoking cessation in European patients with coronary heart disease. Results from the EUROASPIRE IV survey (2018). A registry from the European Society of Cardiology. Int J Cardiol.

[22] Snaterse M, Scholte op Reimer WJM, Dobber J, et al. Smoking cessation after an acute coronary syndrome: immediate quitters are successful quitters. Neth Heart J. 2015;23:600–7. 10.1007/s12471-015-0755-9 PMC4651958 26449241

[23] Snaterse M, Jørstad HT, Minneboo M (2019). Nurse-coordinated referral to a community-based smoking cessation programme in patients with coronary artery disease: results from the RESPONSE-2 study. Eur J Cardiovasc Nursing.

[24] Frederix I, Vanhees L, Dendale P (2015). A review of telerehabilitation for cardiac patients. J Telemed Telecare.

[25] Rawstorn JC, Gant N, Direito A (2016). Telehealth exercise-based cardiac rehabilitation: a systematic review and meta-analysis. Heart.

[26] Kraal JJ, Peek N, Marle MEA-V (2013). Effects and costs of home-based training with telemonitoring guidance in low to moderate risk patients entering cardiac rehabilitation: The FIT@Home study. BMC Cardiovasc Disord.

[27] Maddison R, Pfaeffli L, Whittaker R (2015). A mobile phone intervention increases physical activity in people with cardiovascular disease: Results from the HEART randomized controlled trial. Eur J Prev Cardiol.

[28] Snoek JA, Meindersma EP, Prins LF, et al. The sustained effects of extending cardiac rehabilitation with a six-month telemonitoring and telecoaching programme on fitness, quality of life, cardiovascular risk factors and care utilisation in CAD patients: The TeleCaRe study. J Telemed Telecare. 2019;1357633X19885793. Epub ahead of print. 10.1177/1357633X19885793 31760855

[29] Kraal JJ, Van den Akker-Van Marle ME, Abu-Hanna A (2017). Clinical and cost-effectiveness of home-based cardiac rehabilitation compared to conventional, centre-based cardiac rehabilitation: Results of the FIT@Home study. Eur J Prev Cardiol.

[30] Brouwers RWM, Kraal JJ, Traa SCJ (2017). Effects of cardiac telerehabilitation in patients with coronary artery disease using a personalised patient-centred web application: Protocol for the SmartCare-CAD randomised controlled trial. BMC Cardiovasc Disord.

